# A Randomised Trial Comparing Genotypic and Virtual Phenotypic Interpretation of HIV Drug Resistance: The CREST Study

**DOI:** 10.1371/journal.pctr.0010018

**Published:** 2006-07-28

**Authors:** Gillian Hales, Chris Birch, Suzanne Crowe, Cassy Workman, Jennifer F Hoy, Matthew G Law, Anthony D Kelleher, Douglas Lincoln, Sean Emery

**Affiliations:** 1 National Centre in HIV Epidemiology and Clinical Research, University of New South Wales, Sydney, Australia; 2 Victorian Infectious Diseases Reference Laboratory, North Melbourne, Australia; 3 Burnet Centre, Melbourne, Australia; 4 AIDS Research Initiative, Sydney, Australia; 5 Department of Medicine Alfred Hospital, Monash University, Melbourne, Australia

## Abstract

**Objectives:**

The aim of this study was to compare the efficacy of different HIV drug resistance test reports (genotype and virtual phenotype) in patients who were changing their antiretroviral therapy (ART).

**Design:**

Randomised, open-label trial with 48-week followup.

**Setting:**

The study was conducted in a network of primary healthcare sites in Australia and New Zealand.

**Participants:**

Patients failing current ART with plasma HIV RNA > 2000 copies/mL who wished to change their current ART were eligible. Subjects were required to be > 18 years of age, previously treated with ART, have no intercurrent illnesses requiring active therapy, and to have provided written informed consent.

**Interventions:**

Eligible subjects were randomly assigned to receive a genotype (group A) or genotype plus virtual phenotype (group B) prior to selection of their new antiretroviral regimen.

**Outcome Measures:**

Patient groups were compared for patterns of ART selection and surrogate outcomes (plasma viral load and CD4 counts) on an intention-to-treat basis over a 48-week period.

**Results:**

Three hundred and twenty seven patients completing > one month of followup were included in these analyses. Resistance tests were the primary means by which ART regimens were selected (group A: 64%, group B: 62%; *p* = 0.32). At 48 weeks, there were no significant differences between the groups for mean change from baseline plasma HIV RNA (group A: 0.68 log copies/mL, group B: 0.58 log copies/mL; *p* = 0.23) and mean change from baseline CD4+ cell count (group A: 37 cells/mm^3^, group B: 50 cells/mm^3^; *p* = 0.28).

**Conclusions:**

In the absence of clear demonstrated benefits arising from the use of the virtual phenotype interpretation, this study suggests resistance testing using genotyping linked to a reliable interpretive algorithm is adequate for the management of HIV infection.

## Editorial Commentary

**Background:** Antiretroviral drugs are used to treat patients with HIV infection, with good evidence that they improve prognosis. However, mutations develop in the HIV genome that allow it to evade successful treatment—known as drug resistance—and such mutations are known against every class of antiretroviral drug. Resistance can cause treatment failure and limit the treatment options available. Different types of tests are often used to detect resistance and to work out whether patients should switch to a different drug regimen. Currently, the different types of tests include genotype testing (direct sequencing of genes from virus samples infecting a patient); phenotype testing (a test that assesses the sensitivity of a patient's HIV sample to different drugs), and virtual phenotype testing (a way of interpreting genotype data that estimates the likely viral response to different drugs). The researchers of this study did a trial to find out whether providing an additional virtual phenotype report would be beneficial to patients, as compared with a genotype report alone. The main outcome was HIV viral load after 12 months of treatment, but the researchers also looked at differences in drug regimens prescribed, number of treatment changes in the study, and changes in CD4+ (the type of white blood cell infected by HIV) counts.

**What this trial shows:** The researchers found that the main endpoint of the trial (HIV viral load after 12 months) was no different in patients whose clinicians had received a virtual phenotype report as well as a genotype report, compared with those who had received a genotype report alone. In addition, the average number of drugs prescribed was no different between patients in the two different arms of the trial, and there was no difference in number of drug regimen changes, and no change in immune response (measured using CD4+ cell levels). However, more drugs predicted to be sensitive were prescribed by clinicians who got both a genotype and virtual phenotype report, as compared with clinicians who received only the genotype report.

**Strengths and limitations:** The size of the trial (338 patients recruited) was large enough to properly test the hypothesis that providing a virtual phenotype report as well as a genotype report would result in lower HIV viral loads. Randomization of patients to either intervention ensured that the comparison groups were well-balanced, and the researchers also tested whether selection bias had affected the results (i.e., testing for the possibility that clinicians could predict which intervention participants would receive, and change recruitment into the trial as a result). They found no evidence for selection bias occurring within the trial. However, interpreting the results is difficult because the trial did not directly compare the two different testing platforms, but rather looked at whether providing a virtual phenotype report as well as a genotype report was better than providing a genotype report alone. The investigators also acknowledge that since the trial was conducted, the cutoffs for interpreting genotype information as resistant have been lowered. The findings may therefore not translate precisely to the current situation.

**Contribution to the evidence:** Other cohort studies and clinical trials have shown that patients offered resistance testing respond better to antiretroviral therapy compared with those who were not, but the clinical effectiveness of different resistance testing methods is not known. This study provides additional data on the respective benefits of genotype testing versus genotype plus provision of virtual phenotype. Another trial comparing genotype versus virtual phenotype has also found that the different interpretation methods perform similarly.

## INTRODUCTION

HIV drug resistance was first described three years after the introduction of zidovudine for treatment of HIV infection [1]. Resistance has subsequently been described for each licensed antiretroviral therapy (ART). Some mutations causing resistance to one drug are known to confer cross-resistance to others within the same class [2–4]. The development of resistance to ART significantly reduces drug efficacy and is an important cause of treatment failure.

HIV drug resistance testing is a recommended component of several international ART treatment guidelines [5,6]. Three different methodologies are currently used to evaluate ART susceptibility in clinical HIV isolates; genotype, phenotype, and virtual phenotype. In addition to the different analytical techniques, myriad platforms for the interpretation of laboratory data are available. Genotype testing involves the sequencing of viral genes and the interpretation of mutations detected in the gene sequence. Phenotypic testing directly measures the susceptibility of a clinical HIV isolate to antiretroviral drugs. The virtual phenotype predicts ART susceptibility on the basis of mapping defined point mutations against an extensive database comprising virus gene sequences with defined phenotypic susceptibility to antiretroviral drugs [7].

None of the current methods for resistance testing is ideal. Genotypic testing may not correlate completely with phenotype, requires expert interpretation, and relies heavily on the availability of unvalidated, rules-based algorithms. The technique requires a minimum plasma HIV-1 viral load of approximately 1,000 RNA copies/mL. Phenotypic testing is slow and only available in highly specialised laboratories. Moreover, the relationship between phenotypic inhibitory concentrations (IC50, IC90) and virologic response under continuing drug pressure (the so-called cutoff) requires constant adjustment as more laboratory and clinical information becomes available. Both types of resistance testing may fail to detect minor quasi-species that may have clinical significance. Quality control of genotypic testing for sequence quality, the ability to detect nucleotide mixtures, the interpretation of sequences obtained, and clinician familiarity with the report generated, so that informed treatment choices are made, remain issues of concern.

In general, both genotypic and phenotypic resistance testing has been shown to provide virologic benefit (reduction in viral load and increased proportion of patients with HIV viral load below the limits of detection) compared with control arms [8–11]. Clinicians have been encouraged to incorporate resistance testing as part of their routine clinical assessment of patients prior to initiating and/or changing therapy [12]. There is preliminary evidence that there are costs/benefits arising from the use of resistance testing [13,14]. More recently, in a meta-analysis of published trials, the clinical effectiveness of HIV resistance testing was questioned [15].

While phenotypic and virtual phenotypic testing have been compared [16,17], the relative utilities of virtual phenotypic and genotypic testing have not been assessed. The CREST (can resistance testing enhance selection of therapy) Study was designed as a randomized, prospective evaluation of antiretroviral prescribing and surrogate marker outcome following provision of resistance tests using a genotype alone or a genotype plus virtual phenotype. The study also supported a national quality assurance program for genotype testing (that incorporated a unifying interpretative algorithm) and the development of clinician familiarity with HIV-1 resistance testing at a time when such testing was not routinely available in Australia or New Zealand.

## METHODS

### Participants

HIV-infected patients taking combination ART, with plasma HIV RNA viral load > 2000 copies/mL, who were willing to change therapy and who were more than 18 years of age were eligible to enter the study. Patients were ineligible if they were ART naive, experiencing an acute illness warranting therapeutic management, or judged by the investigator as being unable to understand or comply with the protocol. All patients were required to give written, informed consent prior to entering the study. Institutional ethics approval was granted for all sites involved in the study. Subjects were recruited from a network comprising 41 clinical sites (hospitals, sexual health clinics, and primary care facilities) in Australia and New Zealand.

### Interventions

Patients were randomly assigned to receive a genotypic resistance test (group A) or a genotypic resistance test plus a virtual phenotype (group B).

### Resistance Testing

Each clinical site selected one laboratory to undertake genotype testing for their study participants.

### Genotype Testing

Blood was collected from eligible patients into tubes containing ACD or EDTA anticoagulant at the randomisation visit and sent to the designated laboratory for genotyping of HIV reverse transcriptase (RT) and protease (PR) genes. The laboratories used a variety of platforms to generate the HIV genotype; TRUGENE HIV-1 assay (Visible Genetics, Ontario, Canada), *n* = 1; ViroSeq HIV-1 Genotyping System Version 2 (Applied Biosystems, Foster City, California, United States), *n* = 2; six laboratories used in-house assays with primers that enabled amplification and sequencing of the PR and RT genes. All laboratories participated in an ongoing quality assurance program to ensure that the differing technologies yielded consistent results [18,19]. Genotype results were reported using a trial specific, rules-based algorithm (Table 1) and a common report format. The report identified a given isolate as being sensitive, intermediate, or resistant to a given ART.

**Table 1:**
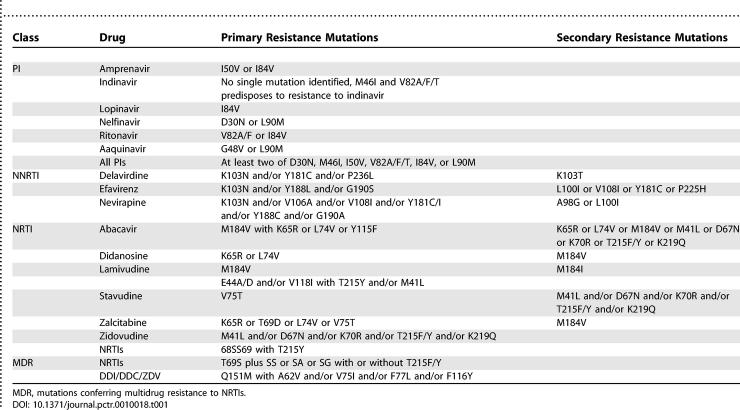
Rules-Based Algorithm for Determining Genotypic Susceptibility to PI, NNRTI, and NRTI in the CREST Substudy

### Virtual Phenotype

The viral RT and PR sequences generated for patients in group B were sent electronically to Virco (Mechelen, Belgium) for generation of a virtual phenotype report. When there were insufficient matches in the database, a Virco rules-based interpretation of the sequence was made. At the time the study was performed, the VircoGEN II platform employed arbitrary clinical cutoffs for each class of ART (<4-fold, 4–10-fold and >10-fold changes for NNRTIs, PIs, and NRTIs, respectively). VircoGEN II was the only format of virtual phenotype used for the duration of the study.

### Objectives

Our primary hypothesis was that provision of the virtual phenotype report would result in significant clinical benefit relative to provision of an HIV genotype report alone in terms of changes in plasma HIV RNA over 48 weeks.

We were also interested in patterns of antiretroviral selection and use following receipt of the study resistance test results, changes in CD4+ cell count, and any associations between clinical outcome and a range of baseline factors.

### Outcomes

The primary endpoint was mean change in log plasma HIV–RNA load between the baseline measurement and that obtained 48 weeks later. Secondary endpoints were: the differences between the planned medication and the regimen selected at baseline following provision of the resistance test result; the proportion of patients with undetectable (<400 copies/mL) plasma HIV-RNA load at 48 weeks; the time weighted average change in log plasma HIV–RNA load from baseline; the number of ART treatment changes over duration of study; the change in CD4+ cell count from baseline to 48 weeks; the time-weighted average change in CD4+ cell count from baseline and time to first ART change.

### Sample Size

Based on an estimate of variability in mean change in log plasma HIV-RNA load between baseline and 48 weeks, corresponding to a standard deviation of 1.0 log_10_, 300 patients were required to detect a difference between groups of 0.35 log_10_ with 80% power using a two-sided significance level of 5%.

### Randomization

Randomization was performed using a central telephone randomization office located at NCHECR. The randomization was stratified by site, first or subsequent combination antiretroviral treatment change, and baseline plasma HIV-RNA load (</>10,000 copies/mL). Computer-generated stratified randomization lists were generated using a blocking factor of two. The block size was known only to key staff at the coordinating centre. It was not known by any of the sites. Eligible patients were randomised in equal proportions to receive either an HIV genotype report (group A) or both an HIV genotype report and a virtual phenotype report (VircoGen II—group B).

Investigators recorded the ARV regimen they would next prescribe based on their clinical judgment prior to receiving the resistance report. Demographic, CD4+ cell count, HIV RNA load, CDC classification, ART history, and ART intolerance data were also collected at baseline.

### Treatment Failure on Study

Patients who failed treatment on the study (defined as plasma HIV RNA load > 5,000 copies/mL on two occasions more than two weeks apart after having achieved <400 copies/mL while on the study) could receive an additional HIV resistance test consistent with their original randomisation.

### Statistical Methods

Primary treatment comparisons were performed using a modified intention to treat (MITT) approach, using all randomised patients with baseline and at least one followup visit. Continuous endpoints were analysed using nonparametric rank-sum tests; binary variables using Fisher's Exact tests. Time to event endpoints were analysed using survival analysis methods. Secondary treatment comparisons were also performed using a strict intention to treat (SITT) approach, including all patients without data as failures, joint worst outcomes, or events at time zero, as appropriate. SITT *p*-values are presented in addition to MITT results.

Two prespecified subgroup analyses were performed—according to number of previous antiretroviral combinations received (≤7 versus 8+ combinations), and by number of drugs resistant according to genotype (≤6 versus 7+ resistant drugs). Evidence for subgroup effects were formally assessed by testing for statistically significant interactions between subgroup and randomized treatment group. At the request of the journal, we also performed one further subgroup analysis according to whether patients were the first or second randomized within each randomization block [20]. The aim of this analysis was to try to assess whether the small block size of two adopted in this trial may have led to biased patient allocation, which might be detectable as differing treatment effects according to whether a patient was the first or second randomized in each block.

### Resistance Testing

Baseline resistance profiles were analysed according to randomisation group using the genotype results only. Additionally, a formal comparison of genotype interpretation according to the Crest Algorithm (Table 1) and virtual phenotype (using locally derived sequence data analysed by VircoGen II) was undertaken on group B patients. Resistance profiles were summarised according to the total number of ART to which a patient's virus was classified as resistant, of intermediate resistance, or sensitive, and the proportion of patients whose virus was resistant to all available ART.

The results of resistance testing for each ART were cross-tabulated against the genotype or virtual phenotype. The proportion of genotype or virtual phenotype results that were derived from a rules-based assessment was calculated. The number of drugs against which the virus was classified as sensitive or intermediate by genotype but resistant by virtual phenotype and vice versa was assessed. A score of the overall difference between genotype and virtual phenotype was then calculated.

## RESULTS

### Patient Disposition, Recruitment, and Baseline Characteristics

**Figure 1:**
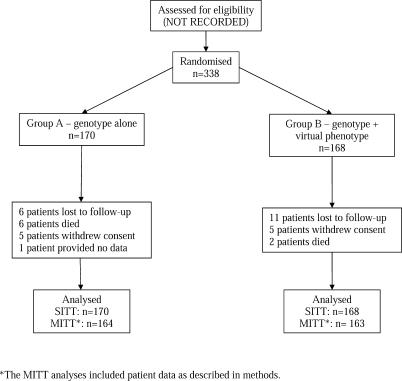
Participant Disposition

A total of 338 patients were randomised into the study between October 2001 and April 2002. Eleven (3.3%) did not have any followup data and were excluded from all analyses. The remaining 327 patients composed the intent-to treat population. Twenty-five patients (7.6%) were lost to followup before 48 weeks, 12 from group A and 13 from group B. These patients were included in analyses using their available data. For participant disposition, please refer to Figure 1. We did not record the numbers of patients screened for the trial who were ineligible.

Baseline patient characteristics were summarised according to randomisation group and are shown in Table 2. The groups were well matched for all AIDS/demographic variables, including gender, prior AIDS, and risk factor. Patients in group A were statistically significantly older than in group B, but the mean difference of two years is not considered clinically relevant. Median plasma HIV RNA at baseline was 16,750 copies/mL in group A and 16,300 copies/mL in group B. Baseline mean CD4+ cell count was 290 cells/mm^3^ in group A and 318 cells/mm^3^ in group B. All patients had previously received nucleoside RT inhibitors (NRTIs), 80% of group A and 79% of group B, had previously received a non-nucleoside RT inhibitor (NNRTI), and 90% in both groups had previously received a protease inhibitor (PI). The mean total number of ARV drugs ever received was eight and the mean duration of ARV treatment was six years in both groups. The mean number of combinations of ARVs used was eight for group A and seven for group B. On average, patients in both treatment groups were known to be intolerant to one ARV drug. The median turnaround times for generation of resistance reports were 33 days (range 16–118 days) for group A and 33 days (range 7–127 days) for group B.

**Table 2:**
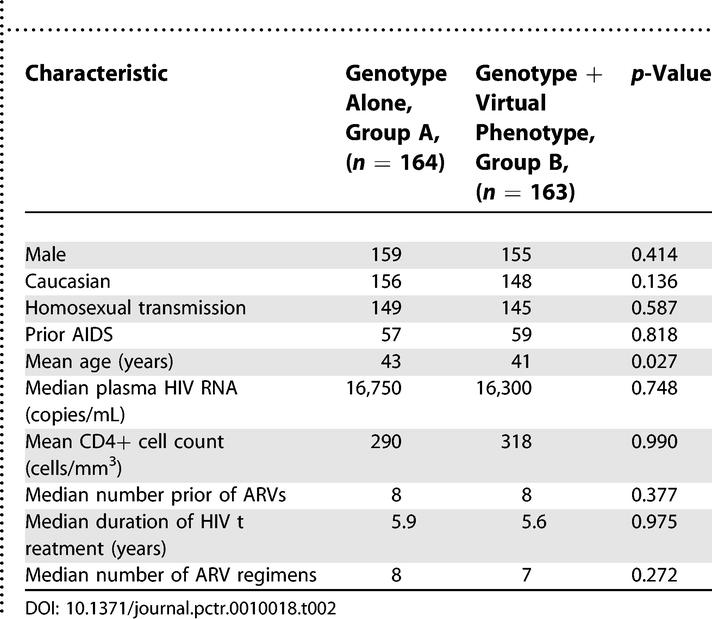
Summary of Patient Baseline Characteristics according to Genotype and Genotype Plus Virtual Phenotype

### Outcomes and Estimation

**Figure 2:**
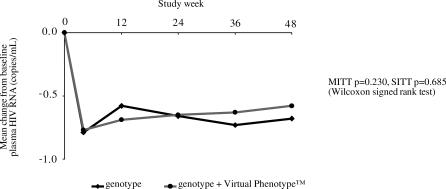
Comparison of Virological Response by Study Arm

After 48 weeks there were no significant differences between the groups for mean change from baseline plasma HIV RNA (group A = −0.68 log copies/mL, group B = −0.58 log copies/mL: MITT *p* = 0.230, SITT *p* = 0.685) (Figure 2). The time-weighted mean viral log change from baseline was −0.61 in group A and −0.58 for those in group B (MITT *p* = 0.876, SITT *p* = 0.952).

A total of 67 patients (46%) in group A and 60 (42%) in group B had undetectable viral loads at week 48 (MITT *p* = 0.553, SITT *p* = 0.502). Mean change from baseline CD4+ cell count at 48 weeks was not significantly different between study groups (group A +37 cells/mm^3^, group B +50 cells/mm^3^; MITT *p* = 0.275, SITT *p* = 0.296). The mean CD4+ weighted change from baseline at week 48 was +24 cells/mm^3^ in group A patients and +39 cells/mm^3^ in group B (MITT *p* = 0.401, SITT *p* = 0.221). Nine (5.5%) patients in group A and seven in group B (4.3%) experienced an AIDS-defining illness during the study. The time to developing an AIDS defining illness was not significantly different between the arms (MITT *p* = 0.625, SITT *p* = 0.579). Eight patients died during the study, six from group A and two from group B.

We performed two prespecified subgroup analyses, in which we examined our study endpoints in patients with or without extensive prior treatment histories, and with multiple drug resistance point mutations or not. We did not observe any evidence of benefit for either resistance test platform in any of these analyses (unpublished data). The analysis according to randomization order also did not reveal any evidence of treatment effects. In patients randomized first within each block, mean log viral load decreases at 48 weeks were −0.70 logs (SD = 1.14) and −0.62 (SD = 0.97) in genotype and virtual phenotype patients, respectively (*p* = 0.574). In patients randomized second, the mean log decreases were −0.65 (SD = 0.94) and −0.51 (SD = 1.09), respectively, (*p* = 0.144). There was no statistical evidence of an interaction between treatment group and randomization order (*p* = 0.535).

### Ancillary Analyses 

#### ARV prescribing at baseline.

The average number of ARVs in regimens prior to randomisation was three in both groups. The mean duration of the current ARV regimen was 18 months in group A and 20 months in group B. The ARV drug class prescribed at study entry prior to resistance testing is shown in Table 3. Three patients (1.9%) in group A, and none in group B, had evidence of genotypic resistance across all drug classes.

**Table 3:**
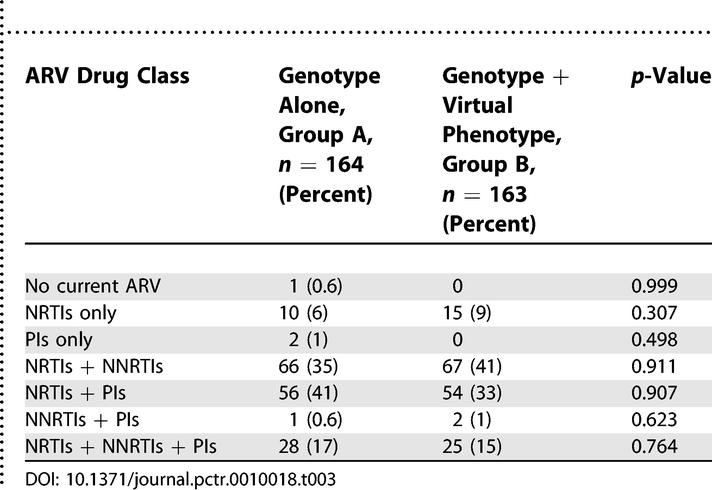
Summary of the ARV Drug Classes Used at Enrollment

Data summarised in Table 4 indicate that the virtual phenotype reported significantly more sensitive ARVs than the genotype platform (MITT and SITT *p* < 0.001). Furthermore, on average clinicians prescribed significantly more sensitive drugs to patients in arm B than in arm A (MITT and SITT and *p* <0.001). There were no apparent differences between study groups for discrepancies between the planned and actual regimen employed. This is despite clear evidence that the overwhelming majority of patients in both groups started a regimen of therapy different from the one planned following receipt and interpretation of either resistance test result.

**Table 4:**
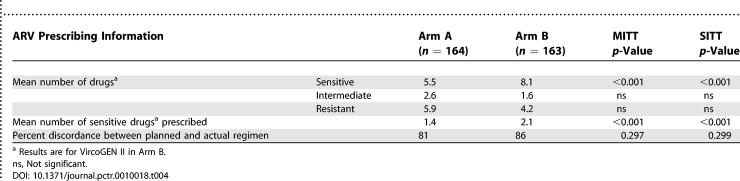
Selected Resistance Test Data and Prescribing Practice Information

The planned ARV regimen was compared with the prescribed ARV regimen following resistance testing. The mean number of ARVs planned and then prescribed in both study groups was three (MITT *p* = 0.311, SITT *p* = 0.342). Thirty two patients (19%) in group A and 22 (14%) in group B were actually prescribed the ARV regimen that had been planned based on best clinical judgment (MITT *p* = 0.180, SITT *p* = 0.182). The reasons cited as being most important for selection of the prescribed ARV regimen were: resistance test result (group A, 64%; group B, 62%; MITT *p* = 0.732, SITT *p* = 0.824), and ARV history (group A: 32%; group B: 28%; MITT *p* = 0.631, SITT *p* = 0.634). The median number of ARV drugs against which plasma virus was sensitive was six in group A and eight in group B. The median number of drugs prescribed against which the virus was sensitive in the resistance test was one in group A and two in group B.

#### ARV changes during the study.

The mean number of changes to prescribed ARV drugs for any reason was three in group A patients and 2.7 in group B (MITT *p* = 0.702, SITT *p* = 0.649). Neither the time to first ARV change nor time to treatment failure were significantly different between the groups (MITT *p* = 0.882, SITT *p* = 0.829, and MITT *p* = 0.382, SITT *p* = 0.557, respectively). The number of protocol-defined treatment failures was 11 (6.7%) in group A and 16 (9.8%) in group B.

#### Resistance results.

**Figure 3:**
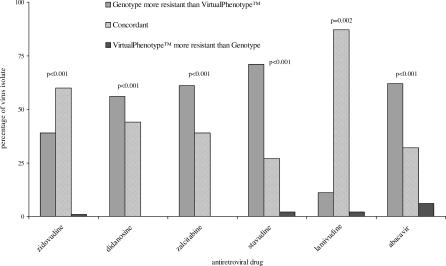
Comparison of Genotype and Virtual Phenotype Reports for NRTIs

**Figure 4:**
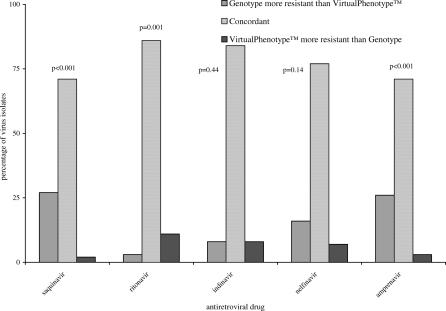
Comparison of Genotype and Virtual Phenotype Reports for PIs

**Figure 5:**
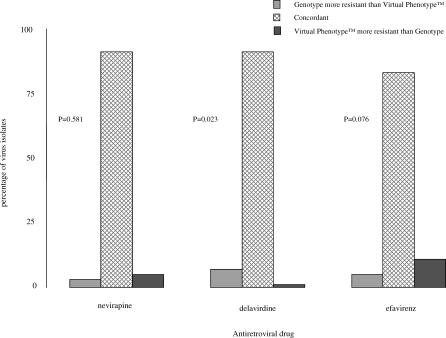
Comparison of Genotype and Virtual Phenotype Reports for NNRTIs

Concordance between the results from genotyping and virtual phenotyping for each drug class were determined (Figures 3–5). There was a significant difference between the susceptibility reported for all NRTIs, with genotype results classifying more patients as harbouring resistant virus than virtual phenotype (*p*-values ≤ 0.002 for all drug classes). NNRTI susceptibility was reported with greater concordance, and only delavirdine resistance was reported more often by genotype than virtual phenotype (*p* = 0.02). There was no significant difference in test concordance for indinavir and nelfinavir, but genotype results reported significantly more clinical isolates resistant to saquinavir (*p* = <0.001) and amprenavir (*p* = <0.001) and virtual phenotype reported significantly more resistance to ritonavir (*p* = 0.001).

## DISCUSSION

### Interpretation

The CREST study was designed to identify whether there was any benefit in providing a virtual phenotype report in addition to a rules-based genotype to assist in the selection of ARV therapy. A no-resistance test control arm was not used because at the time of conducting the trial neither investigators nor patient advocate groups felt there was sufficient clinical equipoise for a control group. Approximately 40% of study patients had viral loads below the level of detection at week 48. Previous studies have also shown the benefit of resistance data when selecting a new ARV regimen [8–11]. The choice of which test to use remains a decision for individual clinicians, who may be influenced by factors such as the test method offered, confidence in the quality of performance of complex laboratory testing procedures, cost, turnaround time, and ease of interpretation of the test report provided.

In this heavily pretreated group of patients, we found no virological or immunological differences at week 48 between these two resistance test platforms.

Subsequent to this study, the clinical cutoffs for resistance employed by virtual phenotyping were lowered from >10-fold to 2.0, 2.0, and 1.8 for didanosine, zalcitabine, and stavudine, respectively. As such it is tempting to dismiss the relevance of the findings. We would argue that the impact of the new cutoffs would be to reduce the number of drugs that are reported back to clinicians as being sensitive and in so doing further reduce the apparent differences between the two virtual phenotype and the genotypic test platforms. This view cannot be informed by data from our trial because the necessary analyses are not possible.

### Generalizability

It is reasonably well-established that extensive prior use of ART results in multidrug resistant virus and that the utility of drug resistance testing in heavily pretreated patients is questionable because their options are very limited [6,10]. However, we found no evidence to suggest that the two test platforms provided any clear benefit in the predefined patient strata with more or less than eight prior regimens and those with virus with resistance to more or less than six available drugs. This observation is supported by findings from the MuSa Study [21].

Our randomised comparison provided clinicians with either one or two forms of report and interpretation describing the scale and scope of antiretroviral drug resistance in clinical isolates taken from patients in the trial. It is not possible to ascertain with confidence which report carried more weight when clinicians reviewed the results provided for patients in group B. Consequently there may be concern that many decisions regarding future treatment in that group were made on the basis of interpretations provided by the genotype result alone. However, our data clearly show that virtual phenotype results provided clinicians and their patients with significantly more treatment options and this resulted in significantly more apparently active drugs being prescribed for patients enrolled in group B. As such, we contend that for the most part our randomised comparison maintained integrity and that the results should be viewed as valid and robust comparisons. Of some importance in this regard is the fact that it is not possible to exclude an interpretation of genotype from the virtual phenotype report since the observed point mutations for a given sample are an integral part of that report.

A majority of clinical HIV isolates in each study arm had at least one ARV with either intermediate or full sensitivity. However, clinician prescribing was limited as not all of these ARVs could be used. For example, a viable combination regimen could not be constructed from three NNRTI drugs. A further limitation of this study is that the impact of low dose ritonavir, used to inhibit cytochrome P450 isoenzymes and thereby increase the plasma levels of co-administered PIs, was not examined. Further work is needed to interpret resistance results when a boosted PI regimen is planned [22,23]. Ongoing refinement and modification of the test platforms does impact upon the generalizability of these results.

CREST resulted in all Australian and New Zealand HIV reference laboratories being able to offer HIV genotype testing and participation in a local quality assurance program. Other outcomes were sharing of expertise, collaboration between different laboratories, and the availability of a standardised genotypic interpretation and reporting format developed and maintained by Australian experts. The study was undertaken in a wide variety of settings and provided investigators with access to HIV resistance test reports. The genotype report was designed to be user friendly for both those experienced and inexperienced in interpreting the results. The provision of a resistance test result significantly impacted the choice of ARVs prescribed across all sites, both in general practice and in specialist hospitals.

### Overall Evidence

Overall, CREST found that HIV resistance test results affected ARV prescribing but there was no additional benefit derived from receiving a virtual phenotype report in addition to a genotype report. This may be due to factors including the quality of the genotype interpretation developed for CREST and the high biological cutoff used in the virtual phenotype analysis. Similar data have been generated in one other trial [21]. Across a range of studies conducted in a diverse patient population there is little data to indicate that any of the myriad interpretation methods offer reliable and predictable benefits. The choice of test platform should probably therefore reflect factors such as availability, cost, and familiarity.

## SUPPORTING INFORMATION

CONSORT Checklist Click here for additional data file. (56 KB DOC)

Trial Protocol Click here for additional data file. (1.0 MB DOC)

## Abbreviations

ART: antiretroviral therapy
CREST: can resistance testing enhance selection of therapy
MITT: modified intention to treat
NNRTI: non-nucleoside RT inhibitors
NRTI: nucleoside RT inhibitors
PR: protease
RT: reverse transcriptase
SITT: strict intention to treat
