# Supplementary material for: A Randomised Trial Comparing Genotypic and Virtual Phenotypic Interpretation of HIV Drug Resistance: The CREST Study
Source: PLoS Clin Trials. 2006 Jul 28;1(3):e18. doi: 10.1371/journal.pctr.0010018 (PMC1523224; doi:10.1371/journal.pctr.0010018)
Supplement: Trial Protocol [file pctr.0010018.sd002.doc]

# Can Resistance Enhance Selection of Therapy (CREST)

##### A randomised, multi-centre study to assess and compare genotypic and virtual phenotypic resistance testing in anti-retroviral experienced, HIV-1 infected individuals with a HIV RNA viral load >2000 copies/ml in whom a change in current antiretroviral therapy is indicated.

Principal Investigators: Participating Laboratory Sites:

Professor Suzanne Crowe Institute Medical and Veterinary Science

MBBS (Hons), FRACP, MD Macfarlane Burnet Centre for Medical Research

Head, AIDS Pathogenesis Research Unit Royal Brisbane Hospital

Consultant Physician in Infectious Diseases Royal Perth Hospital

Alfred Hospital Royal Prince Alfred Hospital

Professor of Medicine St Vincent's Hospital

Monash University. Victorian Infectious Diseases Reference Laboratory

PO Box 254 Westmead Hospital

Fairfield VIC 3078 Auckland Hospital

Ph 03 9282-2194 /2271

Fax 03 9482-6152

Dr Chris Birch Commercial Collaborators:

BSc (Melb), MSc (Melb), PhD (Monash). Virco

Senior Scientist Diagnostic Technology

VIDRL Perkin-Elmer Biosystem

10 Wreckyn Street GlaxoWellcome Australia LTD

North Melbourne 3051 Roche Products Australia PTY LTD

Ph 03 9342 2600 Merck Sharpe and Dohme (Australia) PTY LTD

Fax 03 9342 2665 Boehringer Ingleheim Australia LTD

Bristol-Myers Squibb Australia PTY LTD

Study Implementation:

National Centre in HIV Epidemiology and Clinical Research

Gillian Hales (Study Co-ordinator)

[Ph 02 9331 6320](mailto:Ghales@nchecr.unsw.edu.au)

Fax 02 9332 2485

**Participating Laboratory Sites:**

**Institute Medical and Veterinary Science**

Lesley Rawlings

Frome Road

Adelaide, SA 5000

Ph (08) 8222-3378

Fax 08 8222-3543

[lesley.rawlings@imvs.sa.gov.au](mailto:lesley.rawlings@imvs.sa.gov.au)

**Macfarlane Burnet Centre for Medical Research**

Mandy Dunne

PO Box 254

Fairfield VIC 3078

Ph (03) 9282-2194 /2271

Fax 03-9482-6152

[dunne@burnet.edu.au](mailto:dunne@burnet.edu.au)

**Royal Brisbane Hospital**

Greg Bryson

Queensland Health Pathology Service

Herston Road

Herston, QLD 4006

Ph 07-3253-8048

Fax 07-3636-3623

[brysong@health.qld.gov.au](mailto:brysong@health.qld.gov.au)

**Royal Perth Hospital**

David Sayer

Department of Clinical Immunology

GPO Box X 2213

Perth, WA 6000

Ph 089-224-2899

Fax 08 9224-2920

david@dci.uwa.edu.au

**Royal Prince Alfred Hospital**

Hannan Salem

Building 94, Missenden Road

Camperdown NSW 2050

Ph 02-9515-3585

Fax 02-9351-3205

[hanan@rpamail.cs.nsw.gov.au](mailto:hanan@rpamail.cs.nsw.gov.au)

**St Vincent's Hospital**

Kaz Suzuki

Centre for Immunology

Darlinghurst

NSW 2010

Ph 02 8283 2838

Fax 02 8283 2391

[k.suzuki@cfi.unsw.edu.au](mailto:k.suzuki@cfi.unsw.edu.au)

**VIDRL**

Tracey Middleton

10 Wreckyn Street

North Melbourne 3051

Ph 03 9342 2623

Fax 03 9342 2630

[tracey.middleton@nwhcn.org.au](mailto:tracey.middleton@nwhcn.org.au)

**Westmead Hospital**

Nitin Saksena

Retroviral Genetics Laboratory

Westmead

NSW 2145

Ph 02 9845 6351

Fax 02 9639 7431

[Nitin_saksena@wmi.usyd.edu.au](mailto:Nitin_saksena@wmi.usyd.edu.au)

**Auckland Hospital**

Bryan Schroeder

4^th^ Floor Wallace Block

Grafton Auckland

New Zealand

Ph 61-9-307-6139

Fax 64-9-307-2826

BryanS@ahsl.co.nz

TABLE OF CONTENTS

Eligibility: [6](#__RefHeading___Toc483631027)

Randomisation: [6](#__RefHeading___Toc483631028)

Stratification: [6](#__RefHeading___Toc483631029)

Enrollment: [6](#__RefHeading___Toc483631030)

Primary Study Endpoints [6](#__RefHeading___Toc483631031)

Secondary Study Endpoints: [6](#__RefHeading___Toc483631032)

Additional Analyses [7](#__RefHeading___Toc483631033)

Failed Amplification at Randomisation: [7](#__RefHeading___Toc483631034)

Treatment Change on Study: [7](#__RefHeading___Toc483631035)

Additional Studies: [7](#__RefHeading___Toc483631036)

1. Background and objectives [8](#__RefHeading___Toc483631037)

2. STUDY DESIGN [9](#__RefHeading___Toc483631038)

Randomisation [10](#__RefHeading___Toc483631039)

Stratification [10](#__RefHeading___Toc483631040)

3. Eligibility CRITERIA [10](#__RefHeading___Toc483631041)

Inclusion Criteria [10](#__RefHeading___Toc483631042)

Exclusion Criteria [10](#__RefHeading___Toc483631043)

Patient numbers [11](#__RefHeading___Toc483631044)

Study sites [11](#__RefHeading___Toc483631045)

4. RANDOMISATION PROCEDURE [11](#__RefHeading___Toc483631046)

5. STUDY MEDICATION [11](#__RefHeading___Toc483631047)

6. SCHEDULE OF VISITS [11](#__RefHeading___Toc483631048)

Randomisation Visit [11](#__RefHeading___Toc483631049)

Failed Amplification at Randomisation [11](#__RefHeading___Toc483631050)

Baseline (within 42 days of randomisation) [11](#__RefHeading___Toc483631051)

Week 4 Visit [12](#__RefHeading___Toc483631052)

Standard Study Related Visits [12](#__RefHeading___Toc483631053)

Antiretroviral dose modifications or drug changes [12](#__RefHeading___Toc483631054)

Additional assessments [12](#__RefHeading___Toc483631055)

Study completion (month 12) [13](#__RefHeading___Toc483631056)

Treatment Change on Study [13](#__RefHeading___Toc483631057)

7. PREMATURE WITHDRAWAL [13](#__RefHeading___Toc483631059)

8. SAFETY PARAMETERS [13](#__RefHeading___Toc483631060)

General Parameters [13](#__RefHeading___Toc483631061)

9. ADVERSE EVENT REPORTING [13](#__RefHeading___Toc483631062)

10. SERIOUS ADVERSE EVENTS [13](#__RefHeading___Toc483631063)

11. ETHICAL CONSIDERATIONS [13](#__RefHeading___Toc483631064)

12. STATISTICAL CONSIDERATIONS [13](#__RefHeading___Toc483631065)

Primary Study Endpoints [13](#__RefHeading___Toc483631066)

Secondary Study Endpoints: [14](#__RefHeading___Toc483631067)

Sample Size [14](#__RefHeading___Toc483631068)

Analyses [14](#__RefHeading___Toc483631069)

Baseline Characteristics [14](#__RefHeading___Toc483631070)

Efficacy Analysis [15](#__RefHeading___Toc483631071)

Additional analyses: [15](#__RefHeading___Toc483631072)

13. EARLY STUDY TERMINATION [16](#__RefHeading___Toc483631073)

14. Schema of study related visits [17](#__RefHeading___Toc483631074)

15. SIGNATURE [18](#__RefHeading___Toc483631075)

16. References [19](#__RefHeading___Toc483631076)

**APPENDICES**

Appendix I - Informed Consent

Appendix II - Declaration of Helsinki

Appendix III - Resistance tests preparation

Appendix IV - Plasma & PBMC preparation and storage

Appendix V - Pharmacokinetic Testing

Appendix VI - Compliance Questionnaire

Appendix VII - Genotype Report

**Study summary**

A randomised, multi-centre study to assess and compare genotypic and virtual phenotypic resistance testing in antiretroviral (ARV) experienced, HIV-1 infected individuals with a HIV RNA viral load greater than 2000 copies/ml in whom a change to current ARV therapy is indicated.

## Eligibility:

Patients are eligible to participate if they:

- Are currently receiving ARV therapy

**AND**

- Have an HIV RNA viral load greater than 2000 copies/ml

**AND**

- For whom a new ARV regiment has been planned (but not started) on the basis of best clinical practice.

## Randomisation:

Eligible patients will be randomly assigned in equal proportions to receive one of two formats of report for resistance testing.

Arm 1. Genotypic resistance results with standard laboratory interpretation.

Arm 2. Genotypic resistance results with standard laboratory interpretation + virtual phenotypic resistance results from Virco™.

## Stratification:

Randomisation will be stratified for the following measures:

- Treatment Centre
- Whether or not this is the initial ARV treatment change due to treatment failure.
- HIV RNA viral load (>/< 10,000)

## Enrollment:

A total of 300 subjects will be enrolled into the study over a six-month period and will be followed up for 12 months. Subjects will be enrolled from a range of general practitioner, sexual health clinics and hospital sites across Australia and New Zealand

## Primary Study Endpoints

- Initial virological response and durability of ARV as measured by the change in log plasma HIV-RNA load from baseline at 12 months.

## Secondary Study Endpoints:

- Comparison of planned medication choice prior to receiving resistance results and selected regimen at baseline.
- Proportion of patients with undetectable (<50 copies/ml) plasma HIV-RNA load at 12 months (patients permanently lost to follow-up will be considered to be treatment failures and have detectable viral load).
- Time weighted average change in log plasma HIV-RNA load from baseline.
- Number of ARV treatment changes over duration of study.
- Change in CD4 count from baseline to 12 months, and time weighted average change in CD4 count from baseline.
- Time to first ARV change

## Additional Analyses

- Cost benefit analysis may include estimated changes in life expectancy from decreased viral burden, and cost differences between planned ARV and selected ARV
- Impact of low drug levels on the development of resistance and the therapeutic response
- Impact of poor adherence on the development of resistance and the therapeutic response
- To define new resistance patterns that are associated with treatment failures.

**Interim Analysis**

There will be a formal interim analysis when all randomised patients have completed 6 months of study.

## Failed Amplification at Randomisation:

Patients, at randomisation, for whom amplification fails may undergo a second resistance test.

## Treatment Change on Study:

Any patient changing treatment whilst on study will be assessed by their physician and the likely reasons for change documented. Treatment changes are to be initiated on the basis of clinical practice. A subgroup of virological treatment failures will be eligible for repeat resistance testing, specifically: Patients who fail on their regimen (defined as having an HIV RNA plasma viral load >5000copies/ml, on two occasions more than two weeks apart) who have achieved a HIV RNA plasma viral load <400 copies/ml whilst on this study, may receive further HIV resistance testing. Such patients should be documented as being adequately compliant with their ARV medication and have treatment options available to them. In order to preserve the original, randomisation repeat resistance tests will be made using the same assay system and report the patient was originally randomised too.

## Additional Studies:

- Trough plasma samples will be taken at week 4 to assess PI and NNRTI drug levels
- An assessment of compliance will be made by the investigator at all study visits. The patients will complete a compliance diary for three days, prior to their week 4 visit.
- To determine if the evolution of drug resistance mutations in proviral DNA from patients with undetectable plasma HIV RNA as a predictor treatment failure.

# 1. Background and objectives

HIV drug resistance was first described in 1989^1^, only three years after the introduction of anti-retroviral medication. As in other diseases, the development of resistance to HIV therapy significantly reduces drug efficacy. Drug resistance has been described for all classes of antiretroviral medications and resistance to one drug often confers resistance to other drugs in the same class, thereby giving rise to multi-drug resistance.

HIV drug resistance can be transmitted and has been found in seroconverters and treatment naïve individuals^2^. Drug resistance is one of the major reasons for treatment failure, but can also be caused by a lack of adherence or subinhibitory drug levels.

HIV in an infected individual can exist in a variety of genetically polymorphic forms. As the RNA virus replicates, genetic variability will continue to develop, largely as a consequence of the high rate of virion turnover and the poor fidelity of the virus replicative machinery. At each replication the genome alters from the parental template. The mutated genomes conferring the greatest fitness for survival tend to become the predominant species. In an individual with replicating virus who is also taking antiretroviral therapy, genetic variability conferring resistance to the therapy will select for virus progeny that have a survival advantage, principally reduced sensitivity to antiretroviral therapy.

Techniques to examine the presence of drug- resistant HIV strains are being evaluated with regard to their role in the management of HIV infection. Most commonly, genotypic resistance testing is performed converting viral RNA (or cell associated DNA) to cDNA and then amplifying a target fragment by polymerase chain reaction (PCR) that are then sequenced. Specific point mutations are compared with consensus wild type sequences and are nominally associated with a phenotype that has reduced sensitivity to antiretroviral therapies (principally as single agents). Ideally, based on the resistance test recommendations can be made to improve antiretroviral therapy. Alternatively, phenotypic resistance testing can be used, which is based upon measuring the in vitro 50% or 90% inhibitory concentrations (IC_50_ or IC_90_) of a drug in virus culture systems. This method directly measures the drug concentration necessary to inhibit viral replication.

Two clinical trials have been conducted to date which offer preliminary evidence that the knowledge of HIV-1 drug resistance improves the choice of a new ARV, reducing more efficiently HIV viral load^3,4^. Other studies have indicated that information of baseline resistance profiles can predict virologic responses in individuals changing therapy^5,6^. There is also some preliminary evidence the cost of additional pathology testing (genotyping) can be counterbalanced against additional expenses for drugs^7^.

A number of questions remain to be answered regarding best use of these resistance assays. Equally, clinical practitioners require opportunities to use and evaluate these new technologies. Genotypic testing may not correlate with phenotype and requires expert interpretation. The technique also requires a minimum plasma HIV-1 viral load of at least 1000 copies/ml to be performed. Phenotypic testing is less well developed than genotypic testing, is currently slow and only performed in highly specialised laboratories. Moreover, inhibitory concentrations (IC50, IC90) are poorly defined. Both types of resistance testing can fail to detect minor quasispecies and share a lack of uniformity to the methodology. A third method, virtual phenotyping, performed by Virco™ utilises a library sample which matches the drug history and genotype of the individual to predict phenotype.

The available tests are very encouraging, but have a number limitations. Both genotypic and phenotypic testing of HIV drug resistance require further development and greater standardisation, if they are to have a role in routine patient care.

**This study has a number of objectives:**

- To determine whether resistance testing significantly affects the selection of ARV in patients who are failing therapy.
- To compare whether routine genotypic assessment and interpretation is different from virtual phenotype assessment and interpretation of ARV resistance in patents failing therapy.
- To assess and compare the virological, immunological and clinical impact of resistance testing in HIV infected patients.
- To provide patients and their practitioners access to a new technology.
- To allow laboratories an opportunity to standardise techniques for resistance testing.
- To evaluate the significance of therapeutic drug monitoring and adherence as mechanisms for treatment failures

Primary Study Endpoints

- Initial virological response and durability of ARV as measured by the change in log plasma HIV-RNA load from baseline at 12 months.

Secondary Study Endpoints

- Comparison of planned medication choice prior to receiving resistance results and selected regimen at baseline.
- Proportion of patients with undetectable (<50 copies/ml) plasma HIV-RNA load at 12 months (patient permanently lost to follow-up will be considered to be treatment failures and have detectable viral load).
- Time weighted average change in log plasma HIV-RNA load from baseline.
- Number of ARV treatment changes over duration of study.
- Change in CD4 count from baseline to 12 months, and time weighted average change in CD4 count from baseline.
- Time to first ARV change

## Additional Analyses

- Cost benefit analysis may include estimated changes in life expectancy from decreased viral burden, cost differences between planned ARV and selected ARV
- Impact of low drug levels on the development of resistance and the therapeutic response
- Impact of poor adherence on the development of resistance and the therapeutic response
- To define new resistance patterns that are associated with treatment failures.

**Interim Analysis**

There will be a formal interim analysis when all randomised patients have completed 6 months of study.

## Failed Amplification at Randomisation:

Patients, at randomisation, for whom amplification fails, may undergo a second resistance test.

## Treatment Change on Study:

Any patient changing treatment whilst on study will be assessed by their physician and the likely reasons for change documented. Treatment changes are to be initiated on the basis of clinical practice. A subgroup of virological treatment failures will be eligible for repeat resistance testing, specifically: Patients who fail on their regimen (defined as having an HIV RNA plasma viral load >5000copies/ml, on two occasions more than two weeks apart) who have achieved a HIV RNA plasma viral load <400 copies/ml whilst on this study, may receive further HIV resistance testing. Such patients should be documented as being adequately compliant with their ARV medication and have treatment options available to them. In order to preserve the original, randomisation repeat resistance tests will be made using the same assay system and report the patient was originally randomised too.

## Additional Studies:

- Trough plasma samples will be taken at week 4 to assess PI and NNRTI drug levels
- An assessment of compliance will be made by the investigator at all study visits. The patients will complete a compliance diary for three days, prior to their week 4 visit.
- To determine if the evolution of drug resistance mutations in proviral DNA from patients with undetectable plasma HIV RNA as a predictor treatment failure.

# 2. STUDY DESIGN

A randomised, multi-centre study to assess and compare genotypic and virtual phenotypic resistance testing in HIV-1 infected individuals with a HIV RNA viral load greater than 2000 copies/ml in whom a change to current antiretroviral (ARV) therapy is indicated. The study duration will be 18 months. Recruitment will take place over a 6-month period.

## Randomisation

Eligible patients will be randomly assigned in equal proportions to receive one of two formats of report for resistance testing.

Arm 1. Genotypic resistance results with standard laboratory interpretation.

Arm 2. Genotypic resistance results with standard laboratory interpretation + virtual phenotypic resistance results from Virco™.

## Stratification

Randomisation will be stratified for the following measures:

- Treatment Centre
- Whether or not this is the initial ARV treatment change due to treatment failure.
- HIV RNA viral load (>/< 10,000)

# 3. Eligibility CRITERIA

## Inclusion Criteria

- Age greater than 18 years.
- Written informed consent obtained.
- Currently receiving an unchanged combination ARV therapy for a minimum of six weeks.
- HIV RNA viral load >2000 copies/ml on current therapy.

## Exclusion Criteria

- A current acute illness of sufficient severity to warrant therapeutic management.
- ARV naive patients
- Patients judged by the investigator as being unable to understand or comply with the protocol

## Patient numbers

A total of 300 eligible patients will be enrolled and randomly assigned in equal proportions to each of the treatment arms. Patients will be stratified depending on whether this is their initial or subsequent ARV change with 150 patients stratified to each arm.

## Study sites

Patients will be enrolled through CHRN and NCHECR affiliated general practice, hospital and STD clinics throughout Australia and New Zealand. Patients must fulfill all eligibility criteria in order to participate.

# 4 .RANDOMISATION PROCEDURE

Investigational sites will use a central randomisation facility housed at the offices of the National Centre in HIV Epidemiological and Clinical Research in Sydney. The randomisation code will be generated by computer. The randomisation form will include the clinicians assessment of which ARV they would plan to use next, based upon clinical judgment without use of resistance testing. Randomisation will be stratified according to number of prior ARV combinations received (one or more than one), and by randomisation HIV viral load (>/< 10,000) and study site.

# 5. STUDY MEDICATION

During the course of this study, patients may access any antiretroviral agents that are either licensed, available through expanded access programs or under clinical investigation according to investigator discretion. The study will not limit in any way, treatment choices or clinical practices.

# 6. SCHEDULE OF VISITS

## Randomisation Visit

Patient randomisation visit for the study should occur 28 days prior to the scheduled baseline (entry) visit. Patients should remain on their current regimen until baseline visit.

On this occasion the investigator should ensure that the patient's HIV status and current HIV RNA viral load (must be no more than three months prior to randomisation visit) are clearly documented. The investigator should also ensure that the patient consent procedure is adequately completed and that the patient’s antiviral history is established, details of ARV intolerance noted and the investigator's opinion of why the current regimen has failed is recorded.

- Plasma will be taken for resistance studies (2 x 5.0 ml PPT) and sent to one of the participating laboratories.

The randomisation form should be faxed to NCHECR accompanied by:

- The planned ARV treatment based on best clinical practice without resistance testing.
- The patient's HIV RNA viral load
- Whether this is the patient's first or subsequent ARV change.

## Failed Amplification at Randomisation

Patients, at randomisation, for whom amplification fails, may undergo a second resistance test.

## Baseline (28 days after randomisation)

The following assessments should be undertaken and pathology collected:

- Antiviral history documented
- Prior resistance testing results documented
- Antiviral intolerance documented
- CD4+ T lymphocyte subsets
- HIV plasma RNA (+ method)
- CDC classification
- Selected ARV regimen
- Buffy coat & Plasma storage (2 x 9.0mls ACD)

The patient’s randomisation number should also be documented on this occasion and the prescription for ARV’s given to the patient. The compliance diary should be given to the patient to complete for the three days immediately preceding their week 4 visit. The patient should be asked to make a morning appointment for their week 4 visit and instructed not to take their morning ARV until after blood has been collected for therapeutic blood monitoring.

## Week 4 Visit

- Antiretroviral dose modifications or drug changes
- CD4+ T lymphocyte subsets
- HIV plasma RNA
- Any toxicity recorded which cause a change of ARV therapy.
- Assessment of HIV disease progression
- Assessment of compliance
- Compliance diary collected from patient.
- Trough plasma sample for drug level taken and sent to local laboratory for storage only for patients taking NNRTIs or PIs (2 x 6.0 ml lithium heparin tubes)

## Standard Study Related Visits

The following assessments should be undertaken at the subsequent on study visits conducted at months 3, 6, 9, and 12, in keeping with routine standard of care.

## Antiretroviral dose modifications or drug changes

- CD4+ T lymphocyte subsets
- HIV plasma RNA
- Any toxicity recorded which cause a change of ARV therapy.
- Assessment of HIV disease progression
- Assessment of compliance
- Buffy coat & storage (2 x 9.0mls ACD)

## Additional assessments

- Protease (codons 1-99) and reverse transcriptase sequences (codons 1-as far as assessed) will be collected in a central database for an analysis of the reason of treatment failures to the previous and current drug regimen. Electronic transfer of this data will be made from laboratories directly to NCHECR.
- Drug levels (trough) for protease inhibitors and NNRTIs will be determined at week 4 to evaluate the impact of individual pharmacokinetics on the treatment response (2 x 6.0ml lithium heparin anticoagulant treated whole blood is taken at the end of the dosage interval (trough level)
- Buffy coat will be stored to be used to determine if the evolution of drug resistance mutations in proviral DNA from patients with undetectable plasma HIV RNA as a predictor treatment failure.

## Study completion (month 12)

Buffy coat and Plasma storage (2 x 9.0mls ACD)

## Treatment Change on Study

Any patient changing treatment whilst on study will be assessed by their physician and the likely reasons for change documented. Patients who fail on their regimen (defined as having an HIV RNA plasma viral load >5000copies/ml, on two occasions more than two weeks apart) who have achieved a HIV RNA plasma viral load <400 copies/ml whilst on this study, may receive further HIV resistance testing. Such patients should be documented as being adequately compliant with their ARV medication and have treatment options available to them. In order to preserve the original, randomisation, for study evaluation, repeat resistance tests will be the same assay system and report the patient was originally randomised too.

Plasma will be taken for resistance studies (2 x 5.0 ml PPT) and sent to one of the participating laboratories. A resistance report will be issued within 28 days to the investigator.

# 7. **PRE**MATURE WITHDRAWAL

Participants, unless they explicitly withdraw consent, will continue to be followed up for the duration of the study, regardless of treatment changes.

# 8. SAFETY PARAMETERS

## General Parameters

Safety will be assessed by symptom directed questions at study visits and also by laboratory evaluations undertaken (see section 15 - Schedule of Visits). All usual standards of care should be applied to participants.

# 9. ADVERSE EVENT REPORTING

Adverse events will be monitored at each visit. Subjects will be asked a non-specific question regarding any event they wish to report. Subjects will be instructed to inform the study physician or study staff of adverse events that may occur at any other time. Only adverse events, which precipitate a change in HIV therapy, need be recorded.

# 10. ETHICAL CONSIDERATIONS

All patients must sign a written informed consent prior to study entry. The study protocol must be approved at all participating sites by an NH & MRC constituted institutional ethics committee. Patients will be supplied with a patient information sheet and informed consent. No individual subject data will be supplied to third parties. The participating investigators and nominated NCHECR representative monitor will be the only people aware of the subject's identity. Subjects have the right to withdraw from the study at any stage. This study will conform to the Declaration of Helsinki 1964 as modified by the 41st World Medical Assembly, Hong Kong (Appendix C).

# 11. STATISTICAL CONSIDERATIONS

## Primary Study Endpoints

- Initial virological response and durability of ARV as measured by the change in log plasma HIV-RNA load from baseline at 12 months.

## Secondary Study Endpoints:

- Comparison of planned medication choice prior to receiving resistance results and selected regimen at baseline.
- Proportion of patients with undetectable (<50 copies/ml) plasma HIV-RNA load at 12 months (patient permanently lost to follow-up will be considered to be treatment failures and have detectable viral load).
- Time weighted average change in log plasma HIV-RNA load from baseline.
- Number of ARV treatment changes over duration of study.
- Change in CD4 count from baseline to 12 months, and time weighted average change in CD4 count from baseline.
- Proportion of patients with detectable (>50 copies/ml) plasma HIV-RNA load at 12 months (patient permanently lost to follow-up will be considered to be treatment failures and have detectable viral load).
- Time weighted average change in log plasma HIV-RNA load from baseline.
- Number of ARV treatment changes over duration of study.
- Change in CD4 count from baseline to 12 months, and time weighted average change in CD4 count from baseline.
- Time to first ARV change

## Sample Size

Based on an estimate of variability in change in log plasma HIV-RNA load from baseline at 12 months corresponding to a standard deviation of 1.0 logs, to detect a difference between groups of 0.5 logs with 80% power using a two-sided significance level of 5% and equal numbers of patients in each group, requires 63 patients per group (four groups). To allow for patients permanently lost to follow-up, and patients whose resistance test is unevaluable, 150 patients will be recruited to each of the two strata (initial or subsequent antiretroviral treatment change) giving 80% power to detect a difference in change in log plasma HIV-RNA load from baseline at 12 months of 0.5 logs.

A combined analysis of both strata will have 98% power to detect a difference between randomised groups in change in log plasma HIV-RNA load from baseline at 12 months of 0.5 logs, and 80% power to detect a difference of 0.35 logs.

The proportion of patients with undetectable viral load at 12 months in the standard genotypic resistance testing group is expected to be 40%. A combined analysis of all 300 patients will also give 80% power to detect an increase in this proportion in the virtual phenotype group to 57% (two-sided significance level of 5%).

## Analyses

Analyses of selected versus planned ARV regimens will be performed when the last patient has completed baseline visit. The final statistical analysis will be performed when all randomised patients have completed 12 months follow-up following their baseline visit, or have permanently withdrawn from the trial.

## Baseline Characteristics

Patient characteristics at baseline will be summarised by randomised treatment group using appropriate statistics. Characteristics to be summarised will include age, sex, HIV exposure category, CD4 count, plasma HIV-RNA viral load, prior AIDS defining illnesses and prior antiretroviral treatment history. There will be no formal comparisons of randomised groups in terms of baseline characteristics.

## Efficacy Analysis

All efficacy analyses will compare the randomised strategies on an intention to treat basis regardless of treatments received during the study, and regardless of whether a patient’s resistance test was evaluable.

Changes in log plasma HIV-RNA load, percentage of patients with detectable viral load, changes in CD4 count, and numbers of antiretroviral treatment changes will be summarised by randomised treatment group at each study visit. Planned antiretroviral treatment change based on treatment history only, and the treatment change actually employed at baseline will also be summarised by randomised group. Summaries will be performed for all patients, and separately for strata defined by initial or subsequent antiretroviral treatment change.

Formal analyses will compare the randomised treatment groups in terms of change in log plasma HIV-RNA load from baseline at 12 months; time weighted average change from baseline in log plasma HIV-RNA load; percentage of patients with detectable plasma HIV-RNA load at 12 months; change in CD4 count from baseline at 12 months, time weighed average change from baseline in CD4 count, planned antiretroviral treatment change on the basis of treatment history only versus treatment change actually employed at baseline, time to first ARV treatment change will be summarised by Kaplan-Mier method and groups compared using Cox regression and total number of antiretroviral treatment changes up to 12 months. Analyses of continuous endpoints will use ANOVA methods, while analysis of categorical endpoints will use logistic regression methods. Differences in outcomes between randomised groups in strata defined by initial or subsequent antiretroviral treatment changes will be formally assessed using tests for interaction between randomised group and strata. If there is consistent evidence of differences in outcomes between randomised groups in strata defined by initial or subsequent antiretroviral treatment change, then all analyses will be compare the randomised groups separately within each strata.

## Additional analyses:

The proportion of patients with new resistance mutations or constant resistance profiles will be compared with regard to trough levels above or below the IC50, IC90.

Based on the questionnaire, the adherence to the drug regimen will be grouped into 3 categories (good, average, poor compliance) and compared among patients with or without new resistance mutations.

Individual resistance mutations and combinations mutations at baseline and at the time of treatment failure will be retrospectively analyzed with the aim to detect new resistance patterns that are associated with treatment failures.

Safety - SAE’S will be summarised by randomised treatment group, and according to antiretroviral treatments received.

Interim analyses - Safety data will be periodically reviewed throughout the trial. There will be one formal analysis of efficacy when all randomised patients have completed six months of study follow-up or have been permanently withdrawn from the study. Efficacy endpoints analysed in the interim will include changes in log viral load and CD4 count, at week 24 from baseline, and time to ARV change. Efficacy and safety data will be summarised, along with relevant data from other clinical studies and will be considered by an independent Data Safety Monitoring Board (DSMB), who will make recommendations regarding the trials conduct. Should any efficacy analyses be statistically significant at the p<0.01 level then the decision may be taken to terminate the study.

# 12. Laboratory Genotypic standardisation.

Genotypic resistance test information will be reported in a standard format (Appendix VII). All sequence data for this study will be interpreted using the Stanford database.

# 13. EARLY STUDY TERMINATION

A patient is free to change treatment at any time or enroll conjointly in other clinical trials. Data should continue to be collected on the patient for the 12 month duration of follow up unless the patient explicitly withdraws consent. Assurance should be given that the decision to withdraw will not prejudice future care for that individual.

##

# Schema of study related visits

|  | **Randomisation**  **28 dyas before baseline** | **Baseline**  **(Entry)** | **Month 1** | **Month 3** | **Month 6** | **Month 9** | **Month 12** | **Treatment failure** |
| --- | --- | --- | --- | --- | --- | --- | --- | --- |
| **Consent** | X |  |  |  |  |  |  |  |
| **Planned ARV regimen** | X |  |  |  |  |  |  |  |
| **Prior resistance testing results** | X |  |  |  |  |  |  |  |
| **History (antiviral)** |  | X |  |  |  |  |  |  |
| **HIV Documentation** | X |  |  |  |  |  |  |  |
| **Randomisation** | X |  |  |  |  |  |  |  |
| **Selected ARV Regimen** |  | X |  |  |  |  |  |  |
| **CD4 + T lymphocyte subsets** |  | X | X | X | X | X | X |  |
| **HIV RNA Viral Load** |  | X | X | X | X | X | X |  |
| **Buffy Coat for storage (9.0 ml ACD)** |  | X |  |  |  |  |  |  |
| **Plasma for storage (9.0ml ACD)** |  | X |  |  |  |  | X |  |
| **Plasma for resistance testing**  **(2 x 5.0ml PPT)** | X |  |  |  |  |  |  | X |
| **Adverse Event Recording** (resulting in ARV change) |  |  | X | X | X | X | X |  |
| **ARV dose modifications or changes** |  | X | X | X | X | X | X |  |
| **Adherence** |  | X | **X + Diary** | X | X | X | X |  |
| **HIV Disease Progression** |  | X | X | X | X | X | X |  |
| **TDM trough levels** ( 2 x 6.0ml lithium heparin) |  |  | X |  |  |  |  |  |

# SIGNATURE

I have thoroughly read and reviewed the study protocol. Having read and understood the requirements and conditions of the study protocol, I agree to perform the clinical study according to the international good clinical practice principles and regulatory authority requirements for source document verification and auditing/inspection of the study.

I understand that changes to the protocol must be made in the form of an amendment which has the prior written approval of the Director of NCHECR.

I understand that any violation of the protocol may lead to early termination of the study.

I agree to the following time schedule: The study will start August 2000 and is foreseen to be completed February 2002*.*

I agree to report to NCHECR, within one working day, of any serious adverse event.

**INVESTIGATOR**: ___________ __________________________

Date Signature

**PRINICPAL INVESTIGATORS:**

___________ __________________________

Date Professor Suzanne Crowe

___________ __________________________

Date Dr Chris Birch

# References

1. Larder BA, Darby G and Richman D. HIV with reduced sensitivity to zidovudine Larder BA, Darby G, Richman D. HIV with reduced sensitivity to zidovudine (AZT) isolated during prolonged therapy. *Science* 1989; **243**:1731-34.
2. Imrie A, Beveridge A, Genn W, et al. Transmission of human immunodeficiency virus type 1 resistant to nevirapine and zidovudine. *The Journal of Infectious Diseases* 1997;**175**:1502-6.
3. Durant J, Clevenbergh P, Halfon P, et al. Drug-resistance genotyping in HIV-1 therapy: the VIRADAPT randomised controlled trial. *Lancet* 1999;**353**:2195-2199.
4. Baxter JD, Mayers DL, Wentworth DN, et al. A pilot study of the short-term effects of antiretroviral management based on plasma genotypic antiretroviral resistance testing (GART) in patients failing antiretroviral therapy. *6^th^ Conference on Retroviruses and Opportunistic Infections* Jan 31-Feb 4. [Abstract LB8]
5. Lorenzi P, Opravil M, Hirschel B, et al. Impact of drug resistance mutations on virologic response to salvage therapy. *AIDS* 1999;**13**:F17-F21.
6. Middleton T, Hales G, Smith D, Birch. Baseline HIV reverse transcriptase and protease mutations in a salvage study. *11^th^ Conference for Australasian Society for HIV Medicine* Dec 9-11 [Abstract OR74]
7. Chaix C, Grenier-Sennelier C, Durant J, et al. Economic evaluation of drug resistance genotyping for the adaptation of treatment in HIV-infected patients in the VIRADAPT study. 39^th^ Interscience Conference on Antimicrobial Agents and Chemotherapy, San Francisco USA, Sept 26-29, 1999

Appendix I

**PATIENT INFORMATION STATEMENT AND CONSENT FORM**

CREST: 8/3/2000

__________________________________________________________________________

A randomised, multi-centre study to assess and compare genotypic and virtual phenotypic resistance testing in antiretroviral treatment experienced HIV-1 infected individuals with a HIV RNA viral load >2000 copies/ml in whom a change in current antiretroviral therapy is indicated.

__________________________________________________________________________

Principal Investigators: Professor Suzanne Crowe

Dr Chris Birch

Contact Person: Gillian Hales

Research Nurse

National Centre of HIV Epidemiology and Clinical Research (NCHECR)

Level 2, 376 Victoria Street

Darlinghurst

NSW 2010

(02) 9331 6320

**Patient Information and Consent Form**

1. You are invited to participate in the research study outlined below.

Background to Research Study

HIV, like many bacteria and viruses, is able to change (mutate) and become resistant to the anti-HIV medications that are available. Once the virus is resistant to medications, they are less effective and this is one of the reasons that anti-HIV treatments fail. Once a treatment is less effective it may be important to change to different therapies. Unfortunately once HIV is resistant to one drug it is often resistant to other related drugs and as a result it is difficult to know that a new drug is going to work. There have been a number of research studies which have shown that patients benefit from increased drug effectiveness if the of resistance tests are available when decisions about changing drugs are being made. These studies found that most patients get a better response to new anti-HIV drugs, because drugs are chosen to which the individual's virus is less likely to be resistant. Anti-HIV drug resistance testing is not yet available through the health system in Australia.

Genotyping and phenotyping are two different methods of looking at which drugs an individual's virus is likely to be resistant to. Both of these tests are currently being developed. It is not clear at this time, which is the better of these two tests. A Company in Europe, VIRCO, performs a service called 'virtual phenotyping'. This means they look at genotype results and match it with a phenotype from their records, this is called a 'virtual phenotype'. This study will be testing anti-HIV drug resistance and comparing whether either genotype or virtual phenotypes provide better information in helping make treatment decisions. Please ask your doctor for more information on genotyping and phenotyping if you would like more details on these tests.

**2. Purpose of the Study**

The main purposes of this randomised clinical study are to establish the most effective form of resistance testing, either genotyping or virtual phenotyping. A quality control programme of Australian laboratories performing genotyping will also be part of this study but will not affect you directly.

**3. The Study design**

An ethics committee has examined and approved this study before any participant is allowed to enroll in the study.

A total of approximately 300 subjects will participate from various approved sites in Australia and New Zealand.

You will be randomised (chosen by chance) to either receive:

- Genotyping resistance testing results.

**OR**

- Genotyping and virtual phenotyping resistance testing.

**4. Clinic Visits and Procedures**

If, after reading this information sheet and discussing the trial with your doctor, you decide to take part you will be asked to sign a form confirming that you agree to participate. A maximum of 42 days prior to the start of the study your doctor will carry out a blood test, which will be sent to a laboratory for resistance testing. Your doctor will also fax a copy of your anti-HIV drug history, and the anti-HIV drugs he/she is thinking of changing you too as well as your last HIV viral load to the National Centre for HIV Epidemiology and Clinical Research (NCHECR). This will be sent with your initials and date of birth, not your full name. You will be randomised by the National Centre of HIV Epidemiology and Clinical Research to either have genotyping or genotyping and virtual phenotyping anti-HIV drug resistance testing performed on your blood. You will also be asked to complete a simple assessment of how you are feeling.

Baseline Visit

You will have your current medical condition assessed. You and your doctor will select your new anti-HIV drugs with the assistance of the resistance results. You and your doctor are free to select any anti-HIV drugs that are available.

- Blood will be drawn to determine your haematological and blood chemistry status for safety purposes
- Blood will be drawn to determine your T-cell count and viral load in plasma; some blood will be stored for future tests such as further drug resistance testing.

One month after starting your new treatment

- You will be asked to complete a diary showing when you have taken your anti-HIV medications for the three days prior to this visit, this is to assist the laboratory staff is establishing how much anti-HIV medication should be in your blood. You will asked to come see your doctor in the morning for this visit before taking your morning anti-HIV drugs so that blood can be taken to accurately measure the amount of anti-HIV medication in your blood at it's lowest level.
- You will have your current medical conditions assessed.
- Blood will be drawn to determine your haematological and blood chemistry status for safety purposes
- Blood will be drawn to determine your T-cell count and viral load in plasma; some blood will be stored for future tests such as further drug resistance testing.

Follow up Visits

You will be asked to visit your doctor three, six, nine and 12 months after the baseline visit. You will have your current medical conditions assessed.

- Blood will be drawn to determine your haematological and blood chemistry status for safety purposes
- Blood will be drawn to determine your T-cell count and viral load in plasma; some blood will be stored for future tests such as further drug resistance testing.

Repeat Resistance Testing
If your anti-HIV drugs stop working, whist you are on the study, your doctor may recommend that you have another resistance test performed. This test will be the same type of test, genotyping or virtual phenotyping, that you received at the beginning of the study.

**5. Interaction with concurrent medication**

Always consult your doctor about any other medication or recreational drug that you are taking.

**6. Risks of Participating in the Study**

Anti-HIV drug resistance testing is currently under development and is not perfected as a laboratory test. It is possible that the resistance results you receive may not be completely accurate and may not show the complete range of drug resistance present in the HIV in your body.

**7. Risks of Study Procedures**

As part of this study, you will have your blood drawn (approximately 30mls) at each visit. This procedure is uncomfortable but rarely results in any significant problems. Side effects which have been noted with drawing blood include feeling light-headed or faint, fainting, formation of a blood clot, bruising and/or infection at the site of the needlestick.

**8. Benefits of Participating in the Study**

It is hoped that knowing your anti-HIV drug resistance will help you and your doctor select drugs which will reduce the amount of virus in your body. This information will also be of value to you and to other people infected with HIV in the future.

**9. Alternative Treatments**

Treatments are not being examined in this study. You are free to change to any anti-HIV drugs with your doctor.

**10. Compensation**

Everything will be done to prevent injury that could result from participation in this trial. For medications, which have already been approved by the government authorities to treat HIV disease, normal legal rules on compensation will apply.

**11. Questions about the study**

You may ask the doctor at the study site if you have questions about the study. He/she will do all that is possible to answer your questions and concerns as they arise.

**12. Statement of Subject Rights**

Your participation in this study is entirely voluntary. If you decide not to take part or to withdraw from the study at any time, you will not be penalised nor lose any benefits to which you are otherwise entitled.

Your doctor will have explained the details of this trial to you and answered any questions you may have. You should be satisfied with the information you have been given and had adequate time to consider whether you want to participate. If you decide you would like to take part in this study, you will be asked to sign a consent form. If you do not want to take part, or if you choose to withdraw from the trial at any time, you will continue to receive the best medical care offered by your doctor.

Your doctor may decide to withdraw you from the trial and continue to care for you if, for example, your condition worsens and alternative treatments are thought to be preferable, or if you experience adverse effects during participation in the study. You are encouraged to contact your doctor should you decide not to continue your participation in the study. Your doctor will explain the best way for you to discontinue your participation in the research study.

All information obtained in connection with this study will remain confidential. Your initials and a subject number (assigned to you as part of the trial) will identify you on data collection forms and laboratory results. You will not be identified in any publication or public presentation of data from this study. Authorised personnel will see your medical records from NCHECR and possibly representatives of Government regulatory authorities. However, your records will always be treated as strictly confidential.

If you would like more information about the study, do not hesitate to ask your Doctor. Other people you can ask include Dr Sean Emery (tel. 02-93324648) and Gillian Hales (tel. 02-93316320)

*You will be given a copy of this form to keep.*

**CONSENT FORM**

1. I, .................................................................................. of .........................................

........................................................................, aged ......................................years,

agree to participate as a subject in the experiment described in the subject information statement set out above.

2. I acknowledge that I have read the subject information statement, which explains why I have been selected, the aims of the experiment and the nature and the possible risks of the investigation, and the statement has been explained to me to my satisfaction.

3. Before signing this consent form, I have been given the opportunity of asking any questions relating to any possible physical and mental harm I might suffer as a result of my participation and I have received satisfactory answers.

4. I understand that I can withdraw from the experiment at any time without prejudice to my relationship to …………………../University of New South Wales.

5. I agree that research data gathered from the results of the study may be published, provided that I cannot be identified.

6. I understand that if I have any questions relating to my participation in this research, I may contact Dr ............................ on telephone ................., who will be happy to answer them.

7. I acknowledge receipt of a copy of this Consent Form and the Subject Information Statement.

Signature of subject Please PRINT name Date

Signature of Investigator Please PRINT name Date

Signature of Witness Please PRINT name Date

Nature of Witness

PO**ST STUDY INFORMATION**

To understand the long-term outcomes of having participated in this trial it is important that data continues to be collected on everyone who participated in the trial, even if you withdraw before the end of the study. You are therefore asked to allow your doctor to inform the National Centre of HIV Research and Epidemiology of any serious health problems that occur to you and what medications you are taking at any time in the future. No blood samples or specific visits to your doctor will be necessary. Your confidentiality will be maintained as during the study.

As you may be aware, government health regulations require that many diseases, including AIDS, be reported to the Health Department. Individuals are only identified on the basis of a code consisting of the first two letters of their first and last names, their gender and date of birth. No names or addresses are recorded. As a result, a registry exists of all people diagnosed with AIDS in Australia. It is maintained under full protection of confidentiality by the National Centre for HIV Epidemiology and Clinical Research, Sydney. The registry holds information on the date of diagnosis of AIDS, the AIDS-defining condition(s), and the likely mode of exposure to HIV.

The Australian HIV Observational Database, is to monitor in Australia patterns of HIV disease and its treatment. This will enable us to learn more about the HIV epidemic in Australia, and better target treatment and preventive programs. This database is also maintained under full protection of confidentiality by the National Centre for HIV Epidemiology and Clinical Research, Sydney. Individuals are only identified on the basis of a code consisting of the first two letters of their first and last names, their gender and date of birth. No names or addresses are recorded.

As access is restricted, we seek you consent to use this registry or database, to find out your current health status, should we be unable to locate you through your doctor.

Refusal to sign this consent WILL NOT preclude your entry into the study.

1. I, ..................................................................... of ...................................................

......................................................................., aged ......................... years, agree and fully understand the additional information that is being requested to be collected about me. I understand that I may withdraw my consent for this information to be collected at any time without prejudicing my relationship with my doctor.

___________________________ ___________________________

Signature of participant Please PRINT name Date

___________________________ __________________________

Signature of Investigator(s) Please PRINT name Date

__________________________ __________________________

Signature of witness Please PRINT name Date

__________________________

Nature of Witness

**REVOCATION OF CONSENT**

I hereby wish to WITHDRAW my consent to participate in the research proposal described above and understand that such withdrawal WILL NOT jeopardise any treatment or my relationship with ………………/ University of New South Wales*.*

Signature Date

Please PRINT Name

The section for Revocation of Consent should be forwarded to :

Gillian Hales

National Centre in HIV Epidemiology and Clinical Research

376 Victoria Street

Darlinghurst NSW 2010.

**Appendix II**

**World Medical Association
Declaration of Helsinki**

**Recommendations guiding physicians in
biomedical research involving human subjects**

Adopted by the 18^th^ World Medical Assembly, Helsinki, Finland, June 1864
And amended by the 29^th^ World Medical Assembly, Tokyo, Japan, 1975
35^th^ World Medical Assembly, Venice, Italy, October 1983
and the 41st World Medical Assembly, Hong Kong, September 1989

**Introduction**

It is the mission of the physician to safeguard the health of the people. His or her knowledge and conscience are dedicated to the fulfillment of this mission.

The Declaration of Geneva of the World Medical Association binds the physician with the words, “The health of my patient will be my first consideration”, and the International Code of Medical Ethics declares that, “A physician shall act only in the patient’s interest when providing medical care which might have the effect of weakening the physical and mental condition of the patient”.

The purpose of biomedical research involving human subjects must be to improve diagnostic, therapeutic and prophylactic procedures and the understanding of the aetiology and pathogenesis of disease.

In current medical practice most diagnostic, therapeutic or prophylactic procedures involve hazards. This applies especially to biomedical research.

Medical progress is based on research, which ultimately must rest in part on experimentation involving human subjects.

In the field of biomedical research a fundamental distinction must be recognised between medical research in which the aim is essentially diagnostic or therapeutic for a patient and medical research the essential object of which is purely scientific and without direct diagnostic or therapeutic value to the person subjected to the research, the essential object of which is purely scientific and without direct diagnostic or therapeutic value to the person subjected to the research.

Special caution must be exercised in the conduct of research which may affect the environment; and the welfare of animals used for research must be respected. Because it is essential that the results of laboratory experiments be applied to human beings, to further scientific knowledge and to help suffering humanity, the World Medical Association has prepared the following recommendations as a guide to every physician in biomedical research involving human subjects, they should be kept under review in the future; it must be stressed that the standards drafted are only a guide to physicians all over the world. Physicians are not relieved from criminal, civil and ethical responsibilities under the laws of their own countries.

**I. Basic principles**

1. Biomedical research involving human subjects must conform to generally accepted scientific principles and should be based on adequately performed laboratory and animal experimentation and on a thorough knowledge of the scientific literature.

2. The design and performance of each experimental procedure involving human subjects should be clearly formulated in an experimental protocol which should be transmitted for consideration, comment and guidance to a specially appointed committee independent of the investigator and the sponsor provided that this independent committee is in conformity with the laws and regulations of the country in which the research experiment is performed.

3. Biomedical research involving human subjects should be conducted only by scientifically qualified persons and under the supervision of a clinically competent medical person. The responsibility for the human subject must always rest with a medically qualified person and never rest on the subject of the research, even though the subject has given his or her consent.

4. Biomedical research involving human subjects cannot legitimately be carried out unless the importance of the objective is in proportion to the inherent risk to the subject.

5. Every biomedical research project involving human subjects should be preceded by careful assessment of predictable risks in comparison with foreseeable benefits to the subject or to others. Concern for the interests of the subject must always prevail over the interests of science and society.

6. The right of the research subjects to safeguard his or her integrity must always be respected. Every precaution should be taken to respect the privacy of the subject and to minimise the impact of the study on the subject’s physical and mental integrity and on the personality of the subject.

7. Physicians should abstain from engaging in research projects involving human subjects unless they are satisfied that the hazards involved are believed to be predictable. Physicians should cease any investigation if the hazards are found to outweigh the potential benefits.

8. In publication of the results of his or her research, the physician is obliged to preserve the accuracy of the results. Reports of experimentation not in accordance with the principles laid down in this Declaration should not be accepted for publication.

9. In any research on human beings, each potential subject must be adequately informed of the aims, methods, anticipated benefits and potential hazards of the study and the discomfort it may entail. He or she should be informed that he or she is at liberty to abstain from participation in the study and that he or she is free to withdraw his or her consent to participation at any time. The physician should then obtain the subject’s freely given informed consent, preferably in writing.

10. When obtaining informed consent for the research project the physician should be particularly cautious if the subject is in a dependent relationship to him or her or may consent under duress. In that case the informed consent should be obtained by a physician who is not engaged in the investigation and who is completely independent of this official relationship.

11. In case of legal incompetence, informed consent should be obtained from the legal guardian in accordance with national legislation. Where physical or mental incapacity makes it impossible to obtain informed consent, or when the subject is a minor, permission from the responsible relative replaces that of the subject in accordance with national legislation.

When the minor child is in fact able to give a consent, the minor’s consent must be obtained in addition to the consent of the minor’s legal guardian.

12. The research protocol should always contain a statement of the ethical considerations involved and should indicate that the principles enunciated in the present Declaration are complied with.

**II. Medical research combined with professional care
(clinical research)**

1. In the treatment of the sick person, the physician must be free to use a new diagnostic and therapeutic measure, if in his or her judgement if offers hope of saving life, reestablishing health or alleviating suffering.

2. The potential benefits, hazards and discomforts of a new method should be weighed against the advantages of the best current diagnostic and therapeutic methods.

3. In any medical study, every patient – including those of a control group, if any – should be assured of the best proven diagnostic and therapeutic method.

4. The refusal of the patient to participate in a study must never interfere with the physician-patient relationship.

5. If the physician considers it essential not to obtain informed consent the specific reasons for the proposal should be stated in the experimental protocol for transmission to the independent committee (1,2).

6. The physician can combine medical research with professional care, the objective being the acquisition of new medical knowledge only to the extent that medical research is justified by its potential diagnostic or therapeutic value for the patient.

**III. Non-therapeutic biomedical research involving human subjects (non-clinical biomedical research)**

1. In the purely scientific application of medical research carried out on a human being, it is the duty of the physician to remain the protector of the life and health of the person on whom biomedical research is being carried out.

2. The subjects should be volunteers – either healthy persons, or patients for whom the experimental design is not related to the patient’s illness.

3. The investigator or the investigating team should discontinue the research if in his/her or their judgement it may, if continued, be harmful to the individual.

1. In research on man, the interest of science and society should never take precedence over considerations related to the well-being of the subject.

Appendix III

**Data requirements for central analysis of HIV sequences for
genotypic antiretroviral resistance interpretation**

All participating laboratories will need to standardise the way in which they edit and report sequences to VIRCO to facilitate reproducible, timely and uniform interpretations.

Please find a reference wild type HIV-1 sequence (based on HXB2-D) which may be used as an aid for sequence alignment (this is available electronically if desired – please contact study coordinator for the file)

**Requirements:**

1. All sequence data to be supplied as a TEXT file
2. One sequence file per patient
3. Each sequence file to be clearly identified by unique code per patient per visit
4. All sequences MUST be edited to begin at precisely the same position (see table)
5. All sequences MUST be edited to finish at precisely the same position (see table)

| **Beginning sequence** | **first codon (CCT CAG ….) of HIV-1 PR** |
| --- | --- |
| **End sequence** | **last nucleotide of codon 400 in HIV-1 RT (….GAA ACA)** |

If this is not possible please provide the following information as a minimum

1. All sequence data to be supplied as a TEXT file
2. One sequence file per patient
3. Each sequence file to be clearly identified by unique code per patient per visit
4. Sequence must contain ALL of HIV-1 protease
5. All sequences MUST be edited to begin at precisely the same position (see table)
6. Sequence must contain AT LEAST to codon 240 of HIV-1 RT
7. The sequence must NOT contain gaps (eg. between PR and RT)
8. For laboratories unable to sequence to HIV-1 RT codon 400: ALL sequences MUST finish at the SAME POSITION ( ie. not random)

**Appendix III
Reference sequence**

Reference sequence (based on HXB2-D) which may be used as an aid for sequence alignment (this is available electronically if desired – please contact study coordinator for the file)

start> CCTCAGGTCACTCTTTGGCAACGACCCCTCGTCACAATAAAGATAGGGGG

GCAACTAAAGGAAGCTCTATTAGATACAGGAGCAGATGATACAGTATTAG

AAGAAATGAGTTTGCCAGGAAGATGGAAACCAAAAATGATAGGGGGAATT

GGAGGTTTTATCAAAGTAAGACAGTATGATCAGATACTCATAGAAATCTG

TGGACATAAAGCTATAGGTACAGTATTAGTAGGACCTACACCTGTCAACA

TAATTGGAAGAAATCTGTTGACTCAGATTGGTTGCACTTTAAATTTTCCC

ATTAGCCCTATTGAGACTGTACCAGTAAAATTAAAGCCAGGAATGGATGG

CCCAAAAGTTAAACAATGGCCATTGACAGAAGAAAAAATAAAAGCATTAG

TAGAAATTTGTACAGAGATGGAAAAGGAAGGGAAAATTTCAAAAATTGGG

CCTGAAAATCCATACAATACTCCAGTATTTGCCATAAAGAAAAAAGACAG

TACTAAATGGAGAAAATTAGTAGATTTCAGAGAACTTAATAAGAGAACTC

AAGACTTCTGGGAAGTTCAATTAGGAATACCACATCCCGCAGGGTTAAAA

AAGAAAAAATCAGTAACAGTACTGGATGTGGGTGATGCATATTTTTCAGT

TCCCTTAGATGAAGACTTCAGGAAGTATACTGCATTTACCATACCTAGTA

TAAACAATGAGACACCAGGGATTAGATATCAGTACAATGTGCTTCCACAG

GGATGGAAAGGATCACCAGCAATATTCCAAAGTAGCATGACAAAAATCTT

AGAGCCTTTTAGAAAACAAAATCCAGACATAGTTATCTATCAATACATGG

ATGATTTGTATGTAGGATCTGACTTAGAAATAGGGCAGCATAGAACAAAA

ATAGAGGAGCTGAGACAACATCTGTTGAGGTGGGGACTTACCACACCAGA

CAAAAAACATCAGAAAGAACCTCCATTCCTTTGGATGGGTTATGAACTCC

ATCCTGATAAATGGACAGTACAGCCTATAGTGCTGCCAGAAAAAGACAGC

TGGACTGTCAATGACATACAGAAGTTAGTGGGGAAATTGAATTGGGCAAG

TCAGATTTACCCAGGGATTAAAGTAAGGCAATTATGTAAACTCCTTAGAG

GAACCAAAGCACTAACAGAAGTAATACCACTAACAGAAGAAGCAGAGCTA

GAACTGGCAGAAAACAGAGAGATTCTAAAAGAACCAGTACATGGAGTGTA

TTATGACCCATCAAAAGACTTAATAGCAGAAATACAGAAGCAGGGGCAAG

GCCAATGGACATATCAAATTTATCAAGAGCCATTTAAAAATCTGAAAACA

GGAAAATATGCAAGAATGAGGGGTGCCCACACTAATGATGTAAAACAATT

AACAGAGGCAGTGCAAAAAATAACCACAGAAAGCATAGTAATATGGGGAA

AGACTCCTAAATTTAAACTGCCCATACAAAAGGAAACATGGGAAACA <stop

**APPENDIX IV**

## LABORATORY TECHNICAL PROCEDURE – ‘BUFFY COAT’ DRY CELL PELLET and Plasma preparation

Once collected tubes must be forwarded to the laboratory without delay for processing.

**Specimen requirements**

- 2 x 9.0 ml fresh venous blood collected into acid citrate dextrose (ACD) or EDTA. Fresh blood (within 30 hours) is preferred to ensure high viability of and integrity of leukocytes. Blood should remain at 18-20°C until and during processing.
- 2 x 5.0 ml PPT (pearl top) with whole blood, to be processed within six hours (for Plasma).

**Materials required :**

• Centrifuge capable of spinning primary specimen tubes at 2,000 x g for 15 minutes.

• microcentrifuge capable of >10000 x g with biological hazard sealed rotor suitable for conical 1.5 mL microcentrifuge tubes.

• sterile phosphate buffered saline (PBC) or saline and water.

• Sterile transfer pipettes

(Catalog # 222-1S SAMCO, 1.0 mL individually wrapped sterile transfer pipette.

Crown Scientific 02-9602-7677)

• 1.5 mL sterile polypropylene conical microcentrifuge tubes with ‘o’-rings

(Cat# 72-692 Sarstedt - 08-349-6555)

**Procedure :**

1. Keep 5-6 mL anticoagulant treated whole blood at ambient room temperature until processing.

2. Centrifuge whole blood at 2000 x g at room temperature for 15 minutes.

3. Label two (2) microcentrifuge tubes with permanent marker with patient ID,date of birth, date of collection, and time of collection.

1. White cell (buffy) layer should be visible at the red cell / plasma interface. Using a sterile transfer pipette aspirate the buffy coat layer using a sweeping, circular action to 0.5 – 1 cm below the red cell interface and transfer equally between the two microcentrifuge tubes.
2. Cap tubes and place in microcentrifuge with biological safety sealable rotor and spin >10000 x g for 2 minutes.
3. Resuspend the pellet with 1.0 mL sterile PBS or saline and vortex. Repeat this wash procedure a second time. Note: If heavy red cell contamination is evident, excessive red cells may be lysed by substituting sterile water in the final wash step. Red cell contamination is common in this procedure and will not effect the final product.
4. Aspirate the final wash solution leaving the pellet dry. Freeze pellets at –70·C until shipping is required.
5. Send to central testing laboratory with sufficient dry-ice (solid carbon dioxide) to ensure specimens remain frozen for the duration of transport. Please observe relevant dangerous goods handling requirements of carrier.

**Procedure for PPT Plasma**

- 1. Centrifuge whole blood at 400x g (1800rpm on Sorvall RT6000) for 15 minutes at 20-25°C.
  2. Ship to Resistance laboratory at 4°C, do not freeze

**APPENDIX V**

**PROCEDURE FOR PROCESSING AND STORAGE OF PLASMA FOR
PROTEASE INHIBITOR AND NON-NUCLEOSIDE REVERSE
TRANSCRIPTASE INHIBITOR DRUG LEVELS (PHARMACOKINETICS).**

**Specimen requirements**

•2 x 6.0 mL Lithium heparin anticoagulant treated whole blood.

Do not use gel separator tubes.

Collect morning trough (just before next dose).

Record date/time last dose and date/time sample collected on request form.

Indicate drug(s) to be assayed on request form.

**Materials required**

• 5.0 mL plastic aliquot tubes

• 1.0 mL transfer pipettes

**Procedure**

2.1 Centrifuge blood at 500-600 x g (2500 rpm) for 10 minutes at 20-25°C.

- 1. Remove cell free plasma (within 2 hour of collection)with transfer pipette and aliquot in 3.0 mL volumes in 2 X aliquot tubes. Label vials with indelible ink or -70°C freezer resistant adhesive labels with patient and trial identifiers, visit number, date.

2.3 Note whether specimens are lipaemic or icteric in log in sheets

2.4 Store specimen at -70°C freezer. Complete inventory.

- 1. Specimen will remain stored at site laboratory until the completion of the study unless otherwise specified for batched analyses. Arrangements will be made by NCHECR for the shipment of specimens to the central testing laboratory, Clinical Pharmacology & Toxicology, St. Vincent's Hospital, Sydney.

John Ray
Senior Scientist
Clinical Pharmacology & Toxicology

St. Vincent’s Hospital
Victoria Street Sydney 2010
(02) 9361 2243
(02) 9361 2724 fax
jray@stvincents.com.au

**CREST**

**Date of Visit ___/___/___ Patient Initial ___/___/___ Date of Birth ___/___/___**

Office Use Only

Site #
Randomisation #

**Adherence to medications**

**In the last week how many doses of medication have been missed?***(tick only one of the boxes below)*

**1. One dose or less has been missed **

**2. Two to five doses have been missed **

**3. Six to ten doses have been missed **

**Please record the date, time and dose of the anti HIV medications as you take them, for 3 days before your next appointment with your doctor. This is to help us work out how much anti HIV medication should be in your blood.**

**3 days before appointment**

**Date time drug name dose**

**2 days before appointment**

**Date time drug name dose**

**Day before appointment**

**Date time drug name dose**

*Please make a morning appointment with your doctor. DO NOT take your anti HIV drugs on the morning of your appointment.*

**Appendix VII NAME OF LABORATORY**

**ADDRESS OF LABORATORY**

**ANTIRETROVIRAL SUSCEPTIBILITY**

**HIV GENOTYPE TEST (plasma RNA)**

| *Patient Name*: | | Citizen, JC |
| --- | --- | --- |
| *UR#/Clinic:* | | 123456/Rain St |
| *Date of Birth:* | 01/01/1955 | |
| *Date of specimen:* | 22/06/00 | |
| *Lab. Number:* | 11448 | |

### PROTEASE INHIBITORS

| **DRUG** | **Codons implicated in drug resistance** | |
| --- | --- | --- |
|  | PRIMARY (major)# | SECONDARY (minor)^ |
| Amprenavir | none | M36I |
| Indinavir | none | M36I |
| Nelfinavir | D30N | M36I, N88D |
| Ritonavir | none | M36I |
| Saquinavir | none | M36I |

### REVERSE TRANSCRIPTASE INHIBITORS

| **DRUG** | **Codons implicated in drug resistance** | |
| --- | --- | --- |
|  | PRIMARY (major) | SECONDARY (minor) |
| Abacavir | none | M184V |
| Adefovir | none | none |
| Didanosine | none | M184V |
| Lamivudine | M184V | none |
| Stavudine | none | none |
| Zalcitabine | none | M184V |
| Zidovudine | M41L, T215Y | L210W |
| Delavirdine | none | none |
| Efavirenz | none | none |
| Nevirapine | none | none |

#Primary mutations are generally associated with high level resistance to a drug, ^secondary mutations confer varying levels of resistance. ‘None’ indicates that no mutations currently known to be associated with resistance to that drug were detected. REFERENCES: <http://hivdb.stanford.edu/hiv/>

# For further details, contact Dr. A Smith, ph (01) 234 5678 (Printed: 29/8/26)

COMMENTS: **RESULTS FOR RESEARCH PURPOSES ONLY**

| *Patient Name*: | | Citizen, JC |
| --- | --- | --- |
| *UR#/Clinic:* | | 123456/Rain St |
| *Date of Birth:* | 01/01/1955 | |
| *Date of specimen:* | 22/06/00 | |
| *Lab. Number:* | 11448 | |

| **Mutations at a known resistance site** | **Insertions at a known resistance site** | **Other mutations of patient from consenus** | **Other insertions of patient** |
| --- | --- | --- | --- |
| PROTEASE D30N  N88D | PROTEASE None | PROTEASE I13V  E35D  M36L  G57R  I62V  L63Q  I63V T74S | PROTEASE None |
| REVERSE TRANSCRIPTASE  M41L  M184V  L210W  R211K  T215Y | REVERSE TRANSCRIPTASE  None | REVERSE TRANSCRIPTASE  Q102K  K122E  C162S  K173Q  K238R  V245T  A272P  T286A  V293I  E297K | REVERSE TRANSCRIPTASE  H208RD |
